# The experiences of lecturers in African, Asian and European universities in preparing and delivering blended health research methods courses: a qualitative study

**DOI:** 10.3402/gha.v9.28149

**Published:** 2016-10-06

**Authors:** Myroslava Protsiv, Salla Atkins, for the ARCADE consortium

**Affiliations:** Department of Public Health Sciences, Global Health (IHCAR), Karolinska Institutet, Stockholm, Sweden

**Keywords:** blended learning, global health, e-learning, higher education, doctoral training, research capacity building

## Abstract

**Background:**

Growing demand for Global Health (GH) training and the internationalisation of education requires innovative approaches to training. Blended learning (BL, a form of e-learning combining face-to-face or real-time interaction with computer-assisted learning) is a promising approach for increasing GH research capacity in low- to middle-income countries. Implementing BL, however, requires additional skills and efforts from lecturers. This paper explores lecturers’ views and experiences of delivering BL courses within the context of two north–south collaborative research capacity building projects, ARCADE HSSR and ARCADE RSDH.

**Design:**

We used a qualitative approach to explore the experiences and perceptions of 11 lecturers involved in designing and delivering BL courses collaboratively across university campuses in four countries (South Africa, Uganda, India and Sweden). Data were collected using interviews in person or via Skype. Inductive qualitative content analysis was used.

**Results:**

Participants reported that they felt BL increased access to learning opportunities and made training more flexible and convenient for adult learners, which were major motivations to engage in BL. However, despite eagerness to implement and experiment with BL courses, they lacked capacity and support, and found the task time consuming. They needed to make compromises between course objectives and available technological tools, in the context of poor Internet infrastructure.

**Conclusions:**

BL courses have the potential to build bridges between low- and middle-income contexts and between lecturers and students to meet the demand for GH training. Lecturers were very motivated to try these approaches but encountered obstacles in implementing BL courses. Considerable investments are needed to implement BL and support lecturers in delivering courses.

## Introduction

Demand for Global Health (GH) training, including research training, has increased in both high-income (HI) and low- and middle-income countries (LMIC) (1), partly due to the recognition that countries and their health issues are inevitably interlinked (2) and that health research improves healthcare (3). Insufficient research and management capacity in LMIC (4) affects these countries’ ability to respond to local health challenges which, in turn, may become global challenges (5). Two key areas in which important research capacity gaps exist are (6): social determinants of health (SDH) to understand the broader social factors that impact people's health and health systems and services research (HSS) to design, implement and evaluate innovations in local health systems. These gaps could be addressed through developing new approaches and collaborative networks for research and education across the globe (7–10). The ARCADE HSSR (African Regional Capacity Development for Health Systems and Services Research) and ARCADE RSDH (Asian Regional Capacity Development for Research on Social Determinants of Health) research consortia focused on building this capacity, through implementing collaborative blended learning (BL) courses (www.arcade-project.org).

BL is an approach that integrates online and face-to-face methods of instruction (11) and has potential to build research capacity in LMIC (9). The approach is flexible and can include providing online content for individual learning combined with discussions in the class – an approach known as the ‘flipped classroom’ (12) – or have largely online studies with only an initial face-to-face meeting with a lecturer and peers. BL is considered as effective as traditional learning (13, 14), and also has the advantages of built in flexibility (15), increased self-direction (16) and higher engagement with course material (17). The approach could therefore make a valuable contribution to GH education. An added advantage of using technogy-enhanced teaching methods is linking universities globally and supporting internationalisation (18). However, BL is a fairly new approach in GH education and requires a particular skillset, different roles, and new responsibilities among lecturers who serve as drivers of this innovative education method (19).

ARCADE HSSR and ARCADE RSDH focused on research capacity building in HSS and SDH in Africa and Asia and developed courses on health research methods and more topic-oriented or context-specific courses (for more detail about the projects see (20) in this issue). The consortia experimented with implementing BL courses collaboratively across universities and at the partner universities without collaboration. As this approach was new for most partners it offered a unique opportunity to study how lecturers adapt to BL methods, and what motivates them to take up teaching in this format. To date, there is little published evidence on lecturers’ views and experiences of designing and delivering BL courses (21, 22), particularly lacking ‘rich’ evidence generated from qualitative research (19, 23). As lecturers are key to successful BL courses (17), highlighting their experience can yield evidence for further implementation of BL in GH research education and contribute toward universities’ e-learning strategies. This study aimed to understand the main successes and challenges in preparing and implementing BL from the perspective of lecturers who were part of the ARCADE consortia.

## Methods

We used a qualitative evaluation approach (24) to explore the views of the lecturers who participated in developing or adapting their courses to BL format and who delivered these courses collaboratively across several university sites in 2012–2013. Our research questions were: How did lecturers experience the development and delivery of BL courses? What are the main opportunities and strengths, challenges and obstacles, perceived by the lecturers when developing and delivering BL courses? What should be changed in the course design? What are the technical challenges experienced and the options available for resolving them?

### The courses

Data were collected as a part of a wider evaluation of blended courses in the ARCADE projects. This study involved lecturers who led or participated in the delivery of seven BL courses in 2012–2013. Course participants were postgraduate students, mostly doctoral and master's degree students. The courses were implemented across several university campuses concurrently, forming one virtual classroom that shared a Moodle based e-learning platform (www.moodle.org). The course leader was present in one classroom, and linked to other sites via web conferencing for real-time lectures or discussions. Each site had facilitators available to guide students, answer questions and to provide feedback. Most courses were implemented over 1–2 weeks corresponding to 40 hours of full-time studies, while one was delivered over 13 weeks, part-time (see Table 1 for more detail). The data were collected after four of the courses had been implemented for the first time and two for the second time.

**Table 1:** Overview of blended courses implemented in ARCADE in 2012–2013

Course title (leading university, and other participating institutions)	Short description of the course	Learning outcomes By the end of this course, the student should be able to		Mode of delivery	Assessment methods	N of students
Meta-analysis of diagnostic accuracy tools (MU, KI, SU)	The module on meta-analysis of diagnostic test studies has been designed to learn how to conduct a meta-analysis of diagnostic accuracy study (DAS) – from study design to manuscript preparation.	-	Understand the concepts, meaning and importance of DAS	1 week full-time (1.5 ECTS) Almost 50% synchronous teaching Lectures are recorded during the session and posted on the Moodle platform. The rest of the course focuses on reading articles shared on Moodle and practical training in meta-analysis.	Multiple choice quiz and progressively developed protocol of a meta-analysis of a diagnostic accuracy study	20
		-	Develop a protocol for a meta-analysis study on DAS; able to define the selection criteria for inclusion to meta-analysis			
		-	Carry out a comprehensive literature search of DAS on the selected test			
		-	Manage the data i.e. define variables, collect data, and perform meta-analysis using applicable software			
		-	Prepare a manuscript on a meta-analysis study for publication			
		-	Discuss the limitations/biases and challenges faced with meta-analyses			
Practical approaches to qualitative research (SU, MU, KI)	The course aims to give a general practical basis to researchers wishing to learn qualitative research methods.	-	Understand the fundamental principles behind qualitative research	13 weeks part-time (7.5 ECTS) Students view lectures and read articles on Moodle for 12 weeks and use a discussion forum. The course also includes 1 week of face-to-face skill training at each site focusing on practical skills, such as conducting interviews and focus-group discussions.	Written assignments and participation in online discussions	27
		-	Design a basic qualitative study			
		-	Do fieldwork including doing individual interviews, focus groups and observation			
		-	Data, write a report and critique qualitative research papers			
Introduction to health system research (MU, KI, SU)	The course provides an introduction to health systems research (HSR) methods applied in health systems approach. It gives an overview of basic methods and the ‘state of the art’ in research and evaluation through the review of major completed studies.	-	Define a health system	1 week full-time (1.5 ECTS) The course consists of online lectures, real-time discussions between all participating sites and reading articles.	Progressively developed protocol of a HSR project	8
		-	Describe the components and functions of the health system			
		-	Identify challenges with health systems			
		-	Be familiar with main research methods			
		-	Formulate HSR questions			
Randomised controlled trials (SU, KI, MU)	The course covers the principles of comparative clinical trials in investigating effectiveness, efficacy and safety of treatments, including different types of trials, strength and weakness of each design; ethics; good clinical practice and regulatory requirements; and principles of trial conduct.	-	Critically reflect on the strengths of randomised controlled trials in the evaluation of interventions	The course is given over 10 weeks at SU, and more intensive version (1 week full-time) is offered over the last week at KI, and MU. The students from all three sites participate in real-time lectures and Q&A sessions with the expert.	Progressively developed protocol of an RCT	38
		-	Design a simple randomised controlled trial in written protocol format			
Social determinants of HIV (SJNAHS, UCTH)	The course aims to introduce students to the basic concepts of HIV infection and social factors that determine disease stage and progression.	-	Understand the main aspects of epidemiology including social pathways to disease	2 weeks part-time (1.5 ECTS) self-directed study, readings and video-recorded lectures and 1 face-to-face contact session.	Multiple choice quiz	10
		-	Know the common diagnostic methods and testing policies			
		-	Understand the health seeking and adherence behaviours of diagnosed patients			
Qualitative evaluation in health care (KI, MA, TJMC)	The course focuses on qualitative evaluation methods for health systems and services research. It provides a theoretical and practical orientation to qualitative evaluation.	-	Choose an appropriate qualitative evaluation method for evaluating health systems and services for both their own and their fellow students’ research questions	2 weeks part-time (1.5 ECTS) with 1 week of synchronous real-time online lecturing and recorded lectures available via Moodle; and 1 week of self-directed study and project work to develop an evaluation protocol.	Study protocols developed and multiple choice quiz	12
		-	Know the entire evaluation cycle from research questions to delivering a report			
Improved drug use focusing on rational use of antibiotics (KI, UCTH, TJMC, HMU)	The course introduces the scope and main methods of drug utilisation research. The use of antibiotics and emerging antibiotic resistance are discussed in the course based on examples from countries of different income level and from different parts of the health system (hospitals, pharmacies, traditional healers).	-	Appraise the use of medicinal drugs as one of the main technologies in the health care system, that is also widely used for self-medication/home treatment	1 week full-time studies (1.5 ECTS) The course offers a combination of synchronous real-time interactive lectures and recorded lectures available via Moodle.	Group work to develop an antibiotic awareness campaign	21
		-	Analyse factors affecting drug use, both on macro- and micro-level, and impact of antimicrobial resistance to global health			
		-	Evaluate different methods to increase the rational use of drugs, and in particular antibiotics			

Participating Universities: KI=Karolinska Institutet, Sweden; MU=Makerere University, Uganda; SU=Stellenbosch University, South Africa; MA=Malawi Medical College, Malawi; TJMC=Tongji Medical College of HUST, China; SJNAHS=St. John's National Academy of Health Sciences, India; UCTH=Ujjain Charitable Trust Hospital & Research Centre, India; HMU=Hanoi Medical University, Vietnam.

### Data collection

The framework for course evaluation included mutliple methods and several sources of data: student evaluation surveys, project documents, participant observations (the result of these are reported elsewhere), and lecturers’ interviews (25). The concepts in the framework were informed by relevant literature, internal project meetings and project discussions. The framework considered factors such as how lecturers experienced BL, and main successes and challenges of delivering the course (23), views on the ‘right mix’ of learning activities and on the design of blended courses (26), the capacity of teaching staff to use basic e-learning tools and their perceived ease and usefulness (27). These factors were used to develop questions for the interview guide (see Appendix 1 for the full list of questions). The interview guide was piloted and revised prior to data collection. Interviews were conducted in English, recorded and transcribed.

MP conducted 11 semi-structured interviews with course conveners and lecturers in South Africa, Uganda, India and Sweden. Interviews were conducted in person (*n*=7) or over Skype (*n*=4) in February–March 2014. Sampling was purposive as we wanted to include both lecturers who had been involved in developing the courses and those who had not developed courses but were involved as facilitators, and representing both junior and senior lecturers. Nine participants had developed and led a course and two had facilitated a course at a collaborating site. Five of the participants had more than 10 years of teaching experience, and four had less than 10 years’ experience. We invited participants via email or in person. We stopped adding new interviewees when we reached data saturation.

### Data analysis

We analysed the data using qualitative content analysis as described in Graneheim and Lundman (28). We focused on manifest content, paying attention to differences in responses that may be due to participants’ gender or experience. MP read and re-read the transcribed text of the interviews to familiarise with the content. Thereafter, we detected the meaning units, assigned a code, which were then organised under categories. SA validated the categories and codes with reference to the transcripts. Themes were generated from the finalised categories (see Table 2 for examples of analysis).

**Table 2:** An example of analysis

Meaning unit	Condensed meaning unit		Category		Theme	
OK, the use of the blended courses – the advantage that you can reach so many more people … And the lectures are now recorded and available. And the students can go through them at any point in time. And I think for getting through content it's a really nice method. For qualitative research the difficulty was having … you know, it's not just content … you've got to … There is issue of skills learning, and personal development, and just testing ideas and moving into new paradigm. And I think it's where the methodology was challenged, where blended learning became more difficult … And we tried using the discussion schedules and it worked to some degree, but it was fairly limited. And I think I like the idea of class discussion.	-	Motivation to use blended learning approach is to extend the reach of the course, and allow some flexibility for students to participate	-	Meeting the needs of doctoral students	-	Student needs as main driver for engaging in BL
	-	The delivery of content via recorded video lecture was successful	-	Training aiming to equip young researchers	-	Balancing course content and aims with available tools
	-	Teaching/learning over the Internet about the skills to conduct qualitative research is challenging due to limited interaction				
You need to develop a completely different set of skills, when you are dealing with a blended learning. Because it's not like you have them in the room and you can just nod or say … You really have to give verbal instructions: ‘now please we are going to listen to this site, and now if you don't have any questions, we move to the other site’. And that I think we realised the very moment when we had to deal with this situation. I think it's the same as delivering a lecture [online] is completely different as compared to having everyone in the classroom, because we have to sit in front of the computer, and be aware of the cameras. And, depending on a teaching style, it could work very well, or it could be a little limiting.	-	Lecturers have to develop new skills for lecturing real time and moderating discussion	-	Learning new skills for online teaching	-	Balancing course aims and content with the tools available for BL
	-	Adapting teaching to the online lecturing and dealing with limitations of interaction via web-conferencing	-	Lack of capacity for designing blended learning courses		

### Ethical considerations

Each lecturer gave verbal informed consent. MP emphasised the voluntary nature of participating in the study, confidentiality and their participation not affecting their work. No formal ethical clearance was required for this study as the informants were speaking purely in their professional capacity.

Audio recordings of the interviews were deleted once the analysis was finalised. We used unique numeric identifiers for the quotations, and removed any identifying information such as names, or place of work, from the interview transcripts and the manuscript in order to ensure anonymity and confidentiality.

## Results

The main themes identified were: student needs as the main drivers for engaging in BL; lack of capacity and support; balancing course aims and content with the tools available for BL; and technology as both a source of frustration and as an enabler of effective BL courses. Below we present these themes in more detail.

### Student needs as main driver for engaging in BL

Lecturers discussed their motivations in some detail during the inteviews. While they discussed the new experience of BL as being important for them personally, they were motivated mainly by their desire to cater better for student needs. However, their enthusiasm was decreased by the time investments required to learn about BL and to implement it.

#### BL: an exciting experience, but a steep learning curve, and a time-consuming task

The courses were a new and exciting experience to many, and lecturers found the possibility of extending the reach of the training and including students from different countries rewarding. However, it was also a steep learning curve.

Um, but there was a big or steep learning curve for me. Learning the technology and online platform that was also new for me, so I had to learn quite a lot as well … But, I was quite excited about it, because it was new, and I could immediately see what the benefits are …

*Participant 9, Senior staff, course leader (male)*

Both junior and senior lecturers reported spending considerable time on learning about the approach and preparing their courses, adding to an already heavy workload:

I like the flexibility of online [teaching]. But I wouldn't say it's less time. Because of course, the lectures have to be pre-recorded … [F]or [the course leader] it was much more time, because he had to learn the whole system, and record the lectures, and re-record that didn't work and whatever, all the uploading and everything. Umm. But you have to spend a lot more time on monitoring the discussions, which is something we didn’t do enough, you know.

*Participant 5, Senior staff, co-facilitator, (female)*

Although the participants were positive about BL overall, they reported that the added workload, combined with a lack of additional compensation and incentives and a perceived overall lack of institutional support, reduced their motivation.

#### Meeting the needs of doctoral students

The benefits of BL in creating equal opportunities for students and improving access to learning motivated the participants. They recognised that the needs of adult learners, including busy lifestyles and multiple responsibilities, may prevent them from participating fully in traditional face-to-face classes:

So if they were working during the day they can access materials at night, and if they wanted to access during the day, they could do so. And they could read the material on their own time and do the self-assessments … So I think just the main issue was about giving the students a lot of flexibility to participate in the course. Whether you're working or not, you certainly could participate fully in the course.

*Participant 9, Senior staff, course leader (male)*

Both male and female lecturers were convinced that students appreciated this flexibility:

Why do blended? For all those reasons: better for the environment, good for handicapped people …

People on parental leave. Exactly. People who just don't live in the capital to access that knowledge. I think it's really important.

*Participant 5, Senior staff, co-facilitator (female)*

This participant also highlighted a benefit of increasing the flexibility of her work as a teacher, as she was able to answer questions online when she had time.

Participants also seemed motivated by the challenging nature of the classes. Students’ learning needs, skills and backgrounds were more diverse than in face-to-face classes. Lecturers needed to find a common level of complexity to serve the needs of the entire group:

… [There] was also a mix in training backgrounds. So for example, people in China were more into health policy, and they brought more that view on how they want to influence health policy. Whereas people in India were more biomedical, so they were more into diagnostics. People in Vietnam were more pharmacists, whereas the ones participating in Sweden were more health care professionals with hands-on practice. So, that mix is always what we want to achieve with this course, regardless of the format. So, and that's why interaction face-to-face, like a group work becomes a key part of it.

*Participant 10, Junior Staff, course leader (female)*

The lecturers wanted to meet the needs and expectations of each student, and to ensure the class attained the course's overall learning goals. Some felt that BL catered for this well, by being flexible and accommodating different learning styles. The strong intrinsic motivation to meet these needs, supported by professional values, seemed to override the heavy workload and time investments required by BL.

#### Research capacity development via BL

Another source of motivation was creating training opportunities for young researchers in LMIC, and equipping them with necessary skillsets to be able to conduct rigorous research in their local communities.

They [students from India, China and Vietnam] could participate in the course without travelling here. Because for them of course, it's an investment of time and money, and all this … I mean the alternative for these people … they would have not been able to participate in the course. At least not at this point in time.

*Participant 6, Senior staff, course leader (female)*

Lecturers also thought BL could engage the best teaching staff across institutions, which could benefit students. Reducing travel for experts themselves was also important:

So I think there is a benefit for the students and I think we could improve on that. I think the global efficiency of teaching these courses in multiple places means that if the lecturer can't travel, the lecture can. So no human being. But in Makerere and at Stellenbosch, and at KI we can run these courses, and it happens to be my interest, pragmatic trials, without me travelling. So that is a real capacity development activity across a wider geographic spread than you would otherwise have.

*Participant 8, Senior staff, course leader (male)*

The lecturers also saw BL courses as building capacity by enhancing junior lecturers’ knowledge and teaching skills while designing or facilitating courses.

### Lack of capacity and support

Participants discussed the challenges of capacity and support extensively. Although they were all curious and excited, they had very little or no prior exposure to BL, and therefore few skills to deliver courses.

#### Learning new skills for online teaching

The participants recognised that moderating and engaging students in an online discussion requires different skills than facilitating a discussion in the classroom. They needed to become familiar with the technology and online communication etiquette themselves (and explain it to the students):

You need to develop a completely different set of skills, when you are dealing with a blended learning. Because it's not like you have them in the room and you can just nod or say … You really have to give verbal instructions: “now please we are going to listen to this site, and now if you don't have any questions, we move to the other site”. And that I think we realised that the very moment when we had to deal with this situation. I think it's the same as delivering a lecture [online] is completely different as compared to having everyone in the classroom, because we have to sit in front of the computer, and be aware of the cameras. And, depending on a teaching style, it could work very well, or it could be a little limiting.

*Participant 10, Junior staff, course leader (female)*

#### Lack of capacity for designing BL courses

Having run the blended courses for the first time, the lecturers realised that they needed most assistance with designing learning activities. While being experts in content, the lecturers needed support in using e-learning tools and in getting access to hardware:

I think what we might need to do is to organise a meeting for tutors or teachers to learn how to use the Moodle [e-learning platform] and online issues in teaching. That one is for sure needed. Because as you can realise we are teaching online for the first time. So we are learning as we do.

*Participant 3, Senior staff, course leader (male)*

Views on the support required for BL varied. Some lecturers welcomed opportunities to learn, to be ‘in control’ if problems occured and to be able to ‘fix things’; while others wished for dedicated e-learning support due to the limited amount of time they had available to learn new technologies.

There are two alternatives. You kind of either work with [IT support] and tell him how you want it organised, or you get some training from somewhere to be able to do it yourself. In the end it's easier to do it yourself, but then again you are faced with this is not your full-time job. So, you kind of don't have time to learn it properly, you don't have time to go to the training. So you need somebody who can just sort of do it.

*Participant 1, Junior staff (female)*

Overall, participants agreed that their need for competent and dedicated support from an expert on e-learning courses design was not met. They thought that general Information Technology (IT) services were either not available to them or not sufficiently competent in supporting e-learning.

### Balancing course aims and content with the tools available for BL

Closely linked to the theme of lack of capacity and support, lecturers were faced with trying to match their course objectives with the e-learning tools available.

#### Training aiming to equip young researchers

Lecturers attempted to design their courses to match the aim of increasing research capacity and developing students’ skills with providing access to those not physically present on campus. Most of the courses in ARCADE included learning objectives to do just that – imparting research skills particularly for developing research projects, which they had done before in traditional classroom learning. Most participants attempted to achieve the same with BL:

Exactly. And I think I achieved that. After this [course] people were able to do. The example is Daniel, who published a paper on meta-analysis of gene-Xpert […]. You know, whenever we talk about hands-on, practical training, we think about the classroom … But it is quite interesting that people who were not in the classroom, are actually given the practical skills, were able to do.

*Participant 3, Senior staff, course leader (male)*

Attempting to replicate what was considered useful in classroom learning resulted in lecturers sometimes not using the easiest BL solution. For example, most lecturers still preferred personalised feedback over automated feedback, for example automatically marked multiple choice questions posted on Moodle, since personalised feedback would be more helpful in improving the students’ work. This often increased lecturers’ workloads as student numbers were large.

#### Learning through real-time discussions

Another key issue to consider in designing course content was interaction. Most participants designed courses to be highly interactive, which they considered crucial to teaching GH research. Lecturers with a strong background of ‘constructivist learning’ (29) preferred a dialogue with a student, considering this to result in growth and pushing each others’ boundaries. Because of this focus, they preferred to use synchronous web-conferencing technology to imitate classroom lecturing and discussions.

… I think the content and learning at PhD level comes from discussion with peers, and [is a] sort of ‘communal construction of knowledge’ instead of lecturer telling them what to say or think.

*Participant 1, Junior staff, course leader (female)*

Some participants thought real-time discussions online had more potential than traditional classes:

I think it [BL approach] has more possibilities […] Perhaps that was one other thing that went well and I didn't mention, which would make this blended course having more possibilities, is the opportunity to discuss with the fellow students at other universities. Because we had students, for the live sessions […] from Stellenbosch, we had students at KI. So, the questions would come from any of the students, and I think that was beneficial to all […] When one student asks a question, then the other students are also able to learn from the response to that questions. So I think that is an advantage, which would probably make this kind of course more attractive in future.

*Participant 7, Senior staff, co-facilitator (male)*

Participants also thought that the mix of the professional backgrounds and different contexts resulted in very engaging discussions among the students, which added to learning. However, these interactions were often challenged by technology as unstable Internet connections affected the sound and video quality of the discussions. This approach also resulted in a balancing act: finding the technology that would ‘perform’ to deliver teaching that would match course objectives. One of the alternatives for real-time discussions (synchronous learning activities, requiring all the students connecting at the same time) was using an e-learning platform for asynchronous interaction with students, which allowed students to comment on others’ posts, to replace some of the real-time discussions. These were seen as easier to implement in areas with limited Internet bandwidth, but according to the lecturers resulted in low student participation levels. The lecturers thought being virtually present in the discussion forums could counter this, but felt that this was demanding in terms of time.

### Technology: a frustration and an enabler of effective BL courses

The technology used was an overarching theme in discussions, as BL depends on the availability of appropriate technology and tools. Participants felt that technology was both an enabler and a deterrent of successful courses. The analysis identified two key categories on the effects of technology.

#### Poor Internet connections and IT infrastructure hindered BL

The participants reported that low bandwidth and unreliable Internet connections affected the quality of their lecturing, and their ability to answer questions and give feedback to students during the real-time sessions. Lost connections during lectures distracted from lecture content as lecturers became concerned that everyone was still online:

… [Y]ou had to be very attentive to make sure that the person is still online. So it would distract you to find out if this person is on, is he getting all, so have to say “Hello, Robert, are you still on? Are you with us?”

*Participant 2, Junior staff, course leader (male)*

Participants also thought that poor Internet connections (on university campuses, at home or in the workplace) created barriers to students accessing training material and participation:

If you can't access the materials, and then, you know, you can't go ahead with what you want to do, that could cause a motivated student to just give up, trying to access material. But yes, we did have issue with Internet access and things like that, which I think now have been sorted out. We hope the next course will be better.

*Participant 11, Junior staff, course leader (female)*

The lack of basic IT infrastructure and equipment for BL (microphones, speakers, web-cameras) and low bandwidth Internet, coupled with high cost of Internet access, were discussed as additional obstacles in LMIC (India, Uganda, Malawi).

The Internet facilities are just not adequate. We tried to rely on our institution's Internet, but it didn't work. So I had to pick a man go and buy enough time for a modem [3G], and that's what I used … I think the whole IT area wasn't so nice. Because even the speakers- I had to go and buy speakers. One of my men I sent to buy speakers to put in the room, so that people could hear what I am talking about. So there is a room, where there are supposed to have all those facilities, but they don't work very well.

*Participant 3, Senior staff, course leader (male)*

3G mobile Internet seemed to be a common quick fix solution to run courses, but there was a great expressed need for institutional investments:

I think the problem with Uganda is more their Internet network, technology is not strong at the moment. So that's not the software that we were using, but more the country's technological development. But of course we need to look at how best we can use software that makes it easier for people to access the course. So I guess, from that point of view we do need to review the technology and make sure that we are using the technology or software that makes it easiest for the students.

*Participant 9, Senior staff, course leader (male)*

#### Perceptions of the usefulness of technology: what did technology enable?

Participants agreed that technology had successfully enabled course delivery. However, since there was no prescriptive common format for course design, and staff could choose their e-learning tools, they had varying experiences of these tools. The participants expressed a very specific need for technological solutions that can work with limited bandwidth. After experimenting with several alternatives, it became obvious that the perfect technology is not yet available:

I can't remember now how many technologies we tried … We must have tried half a dozen of these- and none of them are good so far. But I think they are good enough.

*Participant 8, Senior staff, course leader (male)*

The use of e-learning platforms (mostly Moodle), which was considered an easy and user-friendly tool by some, enabled students' access to learning materials, including readings and video lectures, self-assessment exercises, and also provided the option of submitting assignments anytime and anywhere. Other lecturers remained unsure, however, about the added value of the technology. Some commented about their or their students’ preferences for using email instead of e-learning platforms.

## Discussion

Our study revealed that most lecturers involved in ARCADE courses were highly motivated in delivering BL courses, mainly by students' needs and opportunity for capacity building, but were also challenged by adjusting to new roles and ways of working, lack of support (both institutional and IT) and by having to balance their course aims and teaching preferences with the available technology. The lecturers viewed their engagement in BL as an opportunity to experiment and redesign their courses, which they found exciting and challenging (30, 31).

As in other studies, the participants identified the potential of extending training to those for whom traditional face-to-face learning is not a viable option (32, 33), and reported this as a major motivator (23, 34). This is particularly important for GH training programmes (18, 35), where students from LMIC may not find suitable training opportunities locally and therefore go abroad, leaving their families, jobs, and even careers behind. The semi-flexible solutions known as the ‘sandwich model’, where students are trained at universities for periods and return home to work in their home health systems, implemented in north-south partnerships were considered a benchmark for research capacity development and allowed the students to carry on with their coursework and their fieldwork in their home countries (35). However, our study participants discussed BL as having the potential to meet students’ learning needs better, as the approach can suit students' preferences of where and when they want to study as well as enable the use of a combination of various learning modes to enhance their learning experience (34). This could translate into benefits to those working with significant health systems challenges, especiallty those located in remote areas (33, 36), and in this way contribute to LMIC health systems. BL could also adress the equity in course access (37), by encouraging more women to participate in training as some women may have small children whom they do not want to leave to attend training elsewhere. Research should be conducted on the impact of BL on equity in education and health system strengthening.

A key issue in lecturers’ experience was balancing the course content and aims with the technology available. GH research education requires the development of critical thinking, challenging others’ opinions and seeking innovative solutions for GH problems (38). For this, most lecturers considered interactive classes as the best way to meet these goals. Our participants also noted that the presence of multiple classes from various sites, with many students with different backgrounds, was challenging but rewarding, as noted in other attempts to link GH students from the north and south (39). Our participants’ efforts were, however, hampered by lack of reliable Internet access, which may to some degree reduce the students’ learning experience (40) or, as suggested by our participants, jeopardise students’ motivation to study. More effort should be made toward improving infrastructure in LMIC to facilitate taking full advantage of BL courses (41). Further work should be done on ensuring lecturers have user- friendly and useful software options (15, 27), important for the acceptability of BL and for addressing lecturers' concerns about the investments needed of time and energy. Capacity building for lecturers (42–44) could also ensure that they use the best and most appropriate combinations of synchronous and asynchronous methods (26). This capacity building should be addressed in all strategies for institutional engagement in e-learning. Institutional support and resources invested in engaging technical support teams, could contribute to a seamless process of adaptation of e-learning (44).

### Limitations

MP and SA were both part of the ARCADEs project management team. While we made every attempt to step back from the data, our role in the project may have influenced both what the participants said and how the data were analysed. For enhacing the trustworthiness of the study, data were independently analysed, continously discussed, and an external review was conducted. The data for this study were partially collected via Skype and partially in person, which may have implications on the topics discussed and participants’ level of comfort and attention.

Though this study is explorative, and was conducted with few participants, it is one of few explorations of lecturer experiences in developing and conducting BL. All of these courses were conducted with external project funding. Further studies are needed on how lecturers, outside of project funding may experience course development and implementation, and how they are supported by their institutions.

## Conclusions

This study discusses lecturers’ experiences of designing and implementing blended courses in GH research methods, delivered across several university campuses in LMIC and HI countries under the framework of the ARCADE projects. Lecturers were motivated by the benefits of BL: improving access, equity, and building connections, and demotivated by challenges in institutional support and workloads. BL courses can build linkages between universities, lecturers and students, contribute to GH research education and might constitute part of university internationalisation strategies. However, changing to a strategy involving BL courses requires improved infrastructure, strong institutional commitment and support.

## Paper context

Blended learning (BL) combines the best of traditional and e-learning approaches. It offers flexible, engaging, and more enjoyable experiences to students. This study finds that educators are eager to experiment with highly-interactive design of international BL courses, knowing that with BL they can reach more motivated learners and provide individual feedback and support for meeting their learning goals. Further studies are warranted to evaluate impact of BL in building global health research capacity.

## APPENDIX 1

**Interview guide to course instructors**

1. Experience of delivering blended course

- What was the course that you taught?
- What was your experience teaching blended courses in low-and middle-income countries? Or in partnership with these countries?
- What was it like teaching a blended ARCADE course? Please describe.
  - What were the challenges?
  - What were the successes?
  - Did you feel that you were constrained by the blended mode of delivery of the course?
  - Did you feel that BL offered more possibilities than face-to-face teaching?
- What are the differences, in your opinion, of teaching face-to-face courses and blended courses?
  - Prompt: Which do you prefer? Why?
  - Which was more time consuming? Why?
  - What do you think facilitated delivering the course successfully?
  - What is needed to make sure that these courses are run successfully?
  - Did you feel that you had enough training/orientation to be able to adapt face-to-face courses to BL?

2. Getting the ‘right blend’ to achieve the learning outcomes

- What did you think about the courses structure, lengths and mix of face-to-face real-time teaching of the course and self-study materials (specify)?
  - Prompt: Did you feel that 1) the length of the course and 2) proportion of face-to-face and online self-study activities was optimal for achieving intended learning objectives by students?
  - Would you change anything in it? Would you take away content or activities or add any?
  - What would the optimal course look like?

3. Possibilities for feedback

- Do you think you were able to maintain good contact and provide feedback to students during the courses? How was it achieved?

4. Ease of use and perceived usefulness of e-learning tools

- What did you think of the e-learning tools uses (platform, web-conferencing tool (specify which)?
  - Was it easy to use them? Did you or your students experience any problems?
  - Which features of e-learning platform did you use?
- If you preferred to use other means, please explain why.
